# Therapeutic effects of calcium dobesilate on diabetic nephropathy mediated through reduction of expression of PAI-1

**DOI:** 10.3892/etm.2012.755

**Published:** 2012-10-19

**Authors:** XIAOQIAN ZHANG

**Affiliations:** Department of Endocrinology, Shandong Provincial Qianfoshan Hospital, Shandong University, Jinan 250014, P.R. China

**Keywords:** diabetic nephropathy, calcium dobesilate, treatment

## Abstract

The aim of this study was to investigate whether calcium dobesilate (calcium dihydroxy-2,5-benzenesulfonate) may be used to treat diabetic nephropathy. A total of 121 patients with type 2 diabetic nephropathy received calcium dobesilate (500 mg, 3 times a day) for 3 months. The levels of glycated hemoglobin, fasting serum C peptide, triglyceride, total cholesterol, low-density lipoprotein cholesterol, high-density lipoprotein cholesterol, alanine aminotransferase, γ-glutamyl transferase, urea nitrogen, creatinine, hematocrit, plasma viscosity, whole blood reduced viscosity, high, medium and low shear rate whole blood viscosity, fibrinogen, plasminogen activator inhibitor-1 (PAI-1) and endothelin were determined. The urinary albumin excretion rate (UAER) was also determined once a month during the study. The UAER and medium and low shear rate whole blood viscosity were significantly lower in the treated patients. The rate of microalbuminuria normalization was 90%. During the treatment, the UAERs decreased. The results revealed that calcium dobesilate has therapeutic effects on type 2 diabetes patients with microalbuminuria. In addition, the benefit was positively correlated with the calcium dobesilate treatment time. The therapeutic effect may be due to decreases in the levels of PAI-1.

## Introduction

Diabetic nephropathy is a major microvascular complication of diabetes and one of the leading causes of mortality in diabetes patients. Due to the increasing incidence of diabetes and the decreased survival period of patients, diabetes has become the most common cause of end-stage renal disease (ESRD). Of patients with type 1 or type 2 diabetes, 20–30% are diagnosed as having kidney diseases. Globally, approximately 40% of ESRD patients suffer from diabetic nephropathy as the primary disease (1). The cost of diabetic ESRD treatments is increasing. Therefore, effective control of diabetic nephropathy and prevention of ESRD are essential for improving the life quality of patients with diabetes, as well as reducing the economic costs for the patient’s family and society. With the exception of controlling blood sugar levels, blood pressure and protein intake, inhibitors of the angiotensin-converting enzyme and the angiotensin II receptor are the only available therapeutic approaches for treating diabetic nephropathy. Therefore, novel drugs for the treatment of diabetic nephropathy are required.

Calcium dobesilate (calcium dihydroxy-2,5-benzenesulfonate) is a vascular protective compound which was revealed to be the most effective member of a new family of efficient fibroblast growth factor (FGF) inhibitors (2). Calcium dobesilate is a small molecule that has been widely used for treating diabetic retinopathy and chronic venous insufficiency (3,4). However, the mechanism of action of this drug has not been completely elucidated.

It has been demonstrated that calcium dobesilate binds to the heparin-binding domain of FGF-1, thus reducing its activity (2), and it has been demonstrated to be effective for the treatment of rosacea (5) and psoriasis (6), which are clinical manifestations associated with excessive angiogenesis and the overexpression of vascular endothelial growth factor (VEGF).

In the present study, using the pathogenic analysis of diabetic nephropathy and the pharmacological effects of the compound, it was observed that calcium dobesilate had significant effects for the treatment of diabetic nephropathy. Alterations to the associated hematological indicators were observed in patients with type 2 diabetes, in the presence or absence of calcium dobesilate. The effects of calcium dobesilate on urinary albumin levels were also studied. The therapeutic effects were investigated and the mechanisms of the effect of calcium dobesilate on diabetic nephropathy were determined.

## Materials and methods

### Patients

A total of 121 patients were recruited for this study, including 61 males and 60 females with an average age of 45.3 years (range, 33–68 years). The patients had been diagnosed with diabetes for between 3 months and 12 years, with an average of 6.8 years. Prior written and informed consent was obtained from each patient and the study was approved by the ethics review board of Shandong Provincial Qianfoshan Hospital (Jinan, China).

### Treatment

All treated patients received 500 mg of calcium dobesilate 3 times a day. Blood pressure, urinary albumin excretion rate (UAER), liver and kidney function and the levels of blood glucose, blood lipids, glycated hemoglobin (HbA1c), fasting C-peptide, blood viscosity, endothelin (ET) and plasminogen activator inhibitor-1 (PAI-1) were all measured prior to and following treatment. UAER was measured once a month during the treatment. Urinary albumin (ALB) levels were determined using radioimmunoassays. UAER was calculated using the urine volumes and ALB concentrations.

Venous blood was taken between 7 and 8 am after fasting for 10 h and 2 ml of the venous blood serum was separated for the detection of fasting blood glucose (FBG), HbA1c, total cholesterol (CH), triglyceride (TG), high-density lipoproteinprotein cholesterol (HDL-C), low-density lipoprotein cholesterol (LDL-C), alanine aminotransferase (ALT), γ-Valley glutamyl transferase (γ-GT), urea nitrogen (BUN), creatinine (Cr) and fasting C-peptide.

### Detection of blood specimens

FBG was detected using the glucose oxidase method. HbA1c, CH, TG, HDL-C, LDL-C, ALT, γ-GT, BUN and Cr were determined using biochemical methods with a Beckman Coulter (Miami, FL, USA) automatic biochemical analyzer. Fasting C-peptide was detected using a chemiluminescence method with an ACS 180 SE instrument (Bayer, Leverkusen, Germany). Plasma viscosity, high, medium and low shear rate whole blood viscosity were determined using the capillary method with an MVIS-2030 automatic blood rheology analyzer (Chongqing Tianhai, Chongqing, China).

### ELISA

An ELISA kit containing the anti-human PAI-1 antibody and PAI-1 test plasma were purchased from Shanghai Sun Biotechnology Company (Shanghai, China). The presence of the target complex was determined by the color reaction of horseradish peroxidase and the value measured at 492 nm was proportional to the PAI-1 content of the test plasma.

### RNA preparation

Peripheral blood mononuclear cells were isolated from the blood samples of patients by centrifugation, washed with buffers twice and then stored at −80°C for further analysis. Isolation of total RNA was performed using guanidine isothiocyanate (GIT) methods. The purity of the total RNA was assessed using a UV240 spectrophotometer (Daojin company, Shimanishiki, Japan).

### Quantitative reverse transcription (RT)-PCR

Quantitative RT-PCR analysis of the PAI-1 mRNA levels in tissues and cells was performed. The total RNA was harvested from peripheral blood mononuclear cells using the RNeasy kit (Qiagen, Hilden, Germany) according to the manufacturer’s instructions. The RT-PCR experiments were repeated at least 3 times. RNA was reverse transcribed into cDNA using random primers in a Reverse Transcription II system (Promega, Madison, WI, USA) according to the manufacturer’s instructions. Expression of PAI-1 mRNA was quantified by quantitative PCR using an ABI Prism Sequence Detection System (Applied Biosystems, Carlsbad, CA, USA). The primers used in the present study are shown in Table I. An assay reagent containing premixed primers and a VIC™ labeled probe (Applied Biosystems; cat. no. 4310884E) was used to quantify the expression of endogenous GAPDH mRNA. Template-negative and RT-negative conditions were used as controls. Amplification of the endogenous GAPDH cDNA was monitored. The levels (mean values) of the PAI-1 transcripts in patients were calculated.

### Immunoblot assays

Total protein was harvested from the peripheral blood mononuclear cells of the patients. The proteins were separated on 10% SDS-PAGE gels and subjected to immunoblot analysis. The primary antibodies against PAI-1 (∼50 kDa) and β-actin were purchased from Santa Cruz Biotechnology, Inc. (Santa Cruz, CA, USA; anti-PAI-1, cat. no. sc-5297, 1:200 dilution; anti-β-actin, cat. no. sc-130301, 1:10,000 dilution). The secondary antibody used in the present study was goat anti-mouse IgG-HRP (cat. no. sc-2005, 1:10,000 dilution, Santa Cruz Biotechnology, Inc.). Bound antibodies were detected using an ECL system (Pierce Biotechnology, Rockford, IL, USA). The immunoblot experiments were repeated at least 3 times. The mean normalized optical density (OD) of the PAI-1 protein bands relative to the OD of the β-actin band from the same individual was calculated.

### Statistical analysis

Continuous variables were summarized as mean values (mean ± standard error) and compared using the independent sample t-test. P<0.05 was considered to indicate statistically significant differences. All statistical calculations were performed using the SPSS 10.0 software.

## Results

### Calcium dobesilate reduces levels of UAER in patients

**Figure 1:**
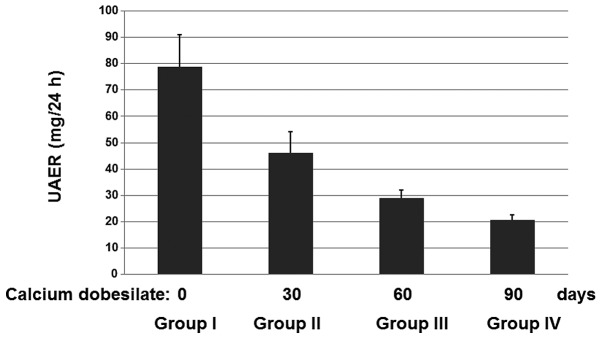
Effects of calcium dobesilate on UAERs in diabetic nephropathy patients. The 121 patients were divided into 4 groups. The patients in group I did not receive calcium dobesilate and served as the control group. The patients in groups II, III and IV received calcium dobesilate for 30, 60 and 90 days, respectively. After 90 days, the UAERs were determined for the patients in each of the 4 groups. UAER, urinary albumin excretion rate.

The diagnosis and staging standard of diabetic nephropathy is based on the UAER. The 121 patients were divided into 4 groups. The patients in group I did not receive calcium dobesilate and served as the control group. The patients in groups II, III and IV received calcium dobesilate for 30, 60 and 90 days, respectively. No adverse drug responses were identified. After 90 days, the UAERs were determined for the patients in each of the 4 groups (Fig. 1). It was observed that the mean UAERs decreased significantly upon treatment with calcium dobesilate compared with the control group. These results suggest that calcium dobesilate is effective for treating diabetic nephropathy.

### Calcium dobesilate significantly reduces the shear rates of whole blood viscosity in patients

As shown in Table II, for the patients receiving calcium dobesilate, PAI-1, ET, plasma viscosity, whole blood reduced viscosity, high, medium shear rate and low shear rate whole blood viscosity and fibrinogen (Fbg) were determined. The medium shear rate and low shear rate whole blood viscosity decreased by a statistically significant amount (Table II).

### Calcium dobesilate did not affect general indicators in patients

The general indicators were also determined for patients treated with or without calcium dobesilate. As shown in Table III, there were no statistically significant effects on the detected levels of FBG, HbA1c, CH, TG, HDL-C, LDL-C, ALT, γ-GT, BUN, Cr, fasting C-peptide, post-prandial 2 h blood glucose (p2hBG), HbA1c, Hct, systolic and diastolic blood pressure (Table III).

### Calcium dobesilate decreases the levels of PAI-1 in patients

**Figure 2:**
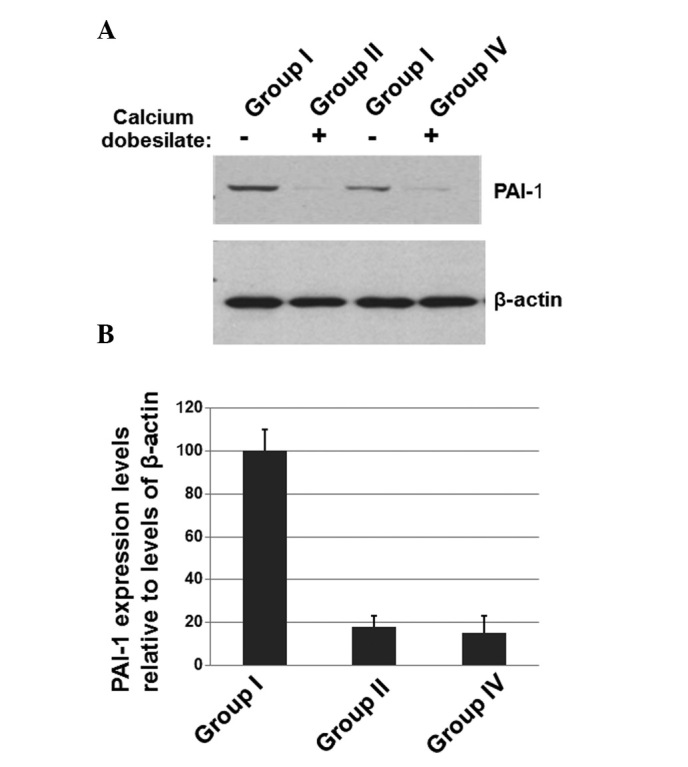
Immunoblots of PAI-1 in patients with diabetic nephropathy. The total protein was harvested, separated on 10% SDS-PAGE gels and subjected to immunoblot analysis. The primary antibodies against PAI-1 (∼50 kDa) and β-actin were anti-PAI-1 (dilution, 1:200) and anti-β-actin (dilution, 1:10,000), respectively. The secondary antibody used in this study was goat anti-mouse IgG-HRP (dilution, 1:10,000). Bound antibodies were detected using an ECL system. (A) Representative blots. (B) The mean normalized OD of PAI-1 protein bands relative to the OD of β-actin bands from each of the 4 groups. PAI-1, plasminogen activator inhibitor-1; OD, optical density.

To determine whether the effects of calcium dobesilate on diabetic nephropathy were associated with the expression of PAI-1, the peripheral blood mononuclear cells were isolated from the blood samples of patients and western blotting was performed. As shown in Fig. 2A, the expression levels of PAI-1 were decreased in patients receiving calcium dobesilate compared with patients who had not received calcium dobesilate. The levels of β-actin were used as a loading control. The mean normalized OD of the PAI-1 protein bands relative to the OD of the β-actin bands was calculated for each of the 4 groups (Fig. 2B). The data for group III are not shown in Fig. 2 since they were similar to those of group II. The results shown in Fig. 2 suggest that calcium dobesilate significantly reduces the expression of PAI-1 which may be associated with the effect of calcium dobesilate on diabetic nephropathy patients.

### Calcium dobesilate decreases the levels of PAI-1 mRNA in patients

**Figure 3:**
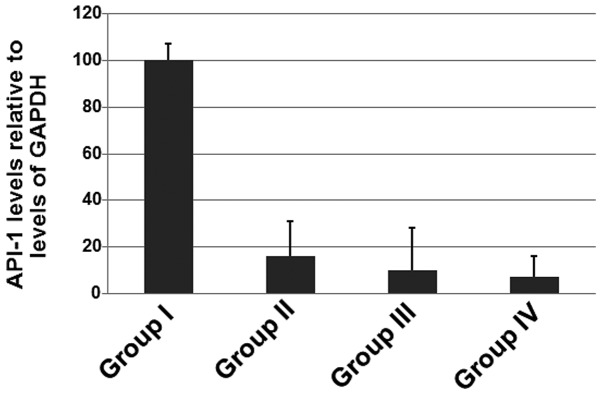
Quantitative RT-PCR analysis of PAI-1 mRNA levels in patients. The total RNA was harvested from the peripheral blood mononuclear cells using an RNeasy kit according to the manufacturer’s instructions. The RT-PCR experiments were repeated at least 3 times. RNA was reverse transcribed into cDNA using random primers in a Reverse Transcription II system according to the manufacturer’s instructions. The expression of PAI-1 mRNA was quantified by quantitative PCR using an ABI Prism Sequence Detection System. Template-negative and RT-negative conditions were used as controls. Amplification of the endogenous GAPDH cDNA was monitored. The levels (mean value) of PAI-1 transcripts in patients were calculated. RT-PCR, reverse-transcription-PCR, PAI-1, plasminogen activator inhibitor-1.

To determine whether calcium dobesilate affects PAI-1 mRNA levels, RT-PCR was performed. As shown in Fig. 3, the expression levels of PAI-1 mRNA were decreased in patients receiving calcium dobesilate compared with those in patients who did not receive calcium dobesilate. The results shown in Fig. 3 suggest that calcium dobesilate significantly reduces the level of PAI-1 mRNA.

## Discussion

The severity of nephropathy is usually defined by the proteinuria level which is closely correlated with kidney damage. It is generally considered that proteinuria increases the glomerular protein filtration channel load, eventually leading to ESRD (7). It was observed in the present study that, following treatment with calcium dobesilate, the UAERs decreased significantly in patients with diabetic nephropathy. The decrease in UAERs was positively correlated with the treatment time, suggesting a therapeutic benefit of this treatment.

In the present study, PAI-1 and Fbg levels were decreased in patients following the administration of calcium dobesilate. There are no previous studies on the effects of calcium dobesilate on PAI-1. However, there are numerous studies concerning the effects of calcium dobesilate on plasma Fbg (8–12) which all reveal decreases in plasma Fbg levels. PAI-1 reduces the inhibition of urokinase and tissue plasminogen activator which activate plasminogen and thus plasmin and eventually alleviates glomerulosclerosis. The decrease in Fbg levels was positively correlated with the reduction of PAI-1 levels.

In the present study, plasma ET levels were decreased following treatment with calcium dobesilate compared with the baseline plasma ET levels. There is a limited number of studies concerning the effect of calcium dobesilate on ET. Zhong and Guo (13) reported that ET levels were significantly decreased in 20 patients with diabetic retinopathy following calcium dobesilate treatment. The reduction effect may be due to the following: i) a reduction in thromboxane A2 (TXA2) levels (14); ii) the removal of oxygen-derived free radicals (15); and iii) a reduction in blood viscosity, thus increasing tissue ischemia, hypoxia and vascular shear stress. Changes in TXA2, free radicals, tissue ischemia and hypoxia and vascular shear stress induce the synthesis and release of ET. This causes the contraction of kidney blood vessels, promotion of cell growth, proliferation and synthesis of extracellular matrix, induction of local inflammation, platelet aggregation and microthrombosis of ET so that a reduction in ET levels may reverse kidney disease. It has also been reported that blood viscosity increases significantly in patients with diabetic nephropathy (16–18).

In the present study, medium shear rate whole blood viscosity and low shear rate whole blood viscosity decreased significantly. Plasma viscosity, whole blood viscosity and high shear rate whole blood viscosity decreased slightly. This demonstrated that the treatment affected aggregation more than the deformability of red blood cells. This effect on blood viscosity was previously only measured in the whole blood or plasma (19–21). In the present study, these indicators were evaluated under various whole blood viscosity shear rates. The effect of calcium dobesilate on diabetic nephropathy may be mediated through a reduction in the expression of PAI-1.

## Figures and Tables

**Table I:** Primers used for RT-PCR.

Primer	Sequence of primer
β-GAPDH F	5′-GTGGGGCGCCCCAGGCACCA-3′
β-GAPDH R	5′-CTCCTTAATGTCACGCACGATTT-3′
PAI-1 F	5′AACATATCTACTAGAAATCTGTT3′
PAI-1 R	5′GCTACTCTACGTCGGCGAGAC3′

F, forward primer; R, reverse primer; PAI-1, plasminogen activator inhibitor-1.

**Table II:** Indicators of patients who were untreated or treated with calcium dobesilate.

Indicator	Untreated (mean ± SE)	Treated (mean ± SE)	P-value
Plasma viscosity (mPas)	1.920±0.962	1.474±0.067	0.081
Whole blood reduced viscosity (mPas)	6.896±0.790	6.467±0.562	0.473
High shear rate whole blood viscosity (mPas)	4.620±0.288	4.407±0.318	0.090
Medium shear rate whole blood viscosity (mPas)	5.474±0.274	5.096±0.374	0.040
Low shear rate whole blood viscosity (mPas)	12.835±0.650	12.101±0.969	0.020
Fbg (g/l)	3.611±0.637	3.064±0.940	0.070
PAI-1 (pg/ml)	69.10±10.600	10.50±11.520	0.062
ET (pg/ml)	68.390±9.562	51.498±8.570	0.060

Fbg, fibrinogen; PAI-1, plasminogen activator inhibitor-1; ET, endothelin. SE, standard error.

**Table III:** General indicators in patients who were untreated or treated with calcium dobesilate.

General indicator	Untreated (mean ± SE)	Treated (mean ± SE)	P-value
FBG (mmol/l)	6.048±0.167	6.183±0.251	0.508
p2hBG (mmol/l)	8.260±0.192	8.330±0.842	0.899
HbA1c (%)	6.520±0.233	6.315±0.322	0.400
Fasting C-peptide (ng/ml)	2.562±1.023	2.130±1.724	0.682
TG (mmol/l)	1.198±0.805	1.394±1.003	0.062
CH (mmol/l)	5.023±1.087	4.807±0.992	0.482
LDL-C (mmol/l)	2.576±0.516	2.502±0.519	0.746
HDL-C (mmol/l)	1.370±0.305	1.385±0.338	0.936
ALT (mmol/l)	25.900±14.449	25.700±14.712	0.963
γ-GT (mmol/l)	20.730±20.195	21.3±15.159	0.876
BUN (mmol/l)	4.546±1.425	4.575±1.932	0.956
Cr (μmol/l)	74.590±19.523	77.300±18.439	0.537
Hct (%)	0.433±0.038	0.413±0.042	0.189
Systolic blood pressure (mmHg)	121.000±6.990	122.500±6.350	0.193
Diastolic blood pressure (mmHg)	74.000±10.490	76.000±2.560	0.309

FBG, fasting blood glucose; p2hBG, post-prandial 2 h blood glucose; HbA1c, glycated hemoglobin; TG, triglyceride; CH, total cholesterol; LDL-C, low-density lipoprotein cholesterol; HDL-C, high-density lipoprotein cholesterol; ALT, alanine aminotransferase; γ-GT, γ-Valley glutamyl transferase; BUN, urea nitrogen; Cr, creatinine; Hct, hematocrit.
